# Physical Activity Patterns and Lifestyle Habits Among Primary Healthcare Workers: A Cross-Sectional Study

**DOI:** 10.3390/ijerph22030323

**Published:** 2025-02-21

**Authors:** Audrey Lehlohonolo Mashita, Mabitsela Hezekiel Mphasha, Linda Skaal

**Affiliations:** 1 Department of Public Health, University of Limpopo, Polokwane 0727, South Africa; mashital@gmail.com; 2 Department of Public Health, Sefako Makgatho University, Ga-Rankuwa 0208, South Africa; linda.skaal@smu.ac.za

**Keywords:** primary healthcare workers, workplace, occupational physical activity, leisure-time physical activity, moderate-intensity activity

## Abstract

Background: Primary healthcare workers (PHCWs) serve as critical contributors to public health, yet their physical activity (PA) patterns and lifestyle habits often reflect the very challenges they address in their patients. The aim of this study is to determine the PA patterns and lifestyle habits among PHCWs in the Lepelle-Nkumpi sub-district of Limpopo Province, South Africa. Methodology: A quantitative, cross-sectional design with stratified random sampling (*n* = 174) was used. A validated, closed-ended questionnaire assessed demographic data and occupational and leisure-time physical activity. Data were analysed using SPSS version 28, with descriptive statistics summarising characteristics. Chi-square tests identified significant associations (*p* < 0.05) between lifestyle scores (poor, good, excellent) and demographic factors. Results: The majority of the participants (53%) reported engaging in shorter walking durations during work, with only 7.5% of the participating engaging in moderate physical activity and 39.7% achieving 30 min or more of activity. Additionally, 59.8% do not use walking or cycling for transportation, while only 27% do so for at least 30 min daily. During leisure time, 33.9% of the participants engage in moderate-to-vigorous physical activities, with 37.9% spending 30 min or more on such activities daily. Nearly two-thirds of the participants (65.5%) had poor lifestyle scores (0–50%), while 23.6% achieved excellent scores (81–100%). Significant associations were found between gender and lifestyle scores (*p* = 0.022). Conclusion: This study reveals critical gaps in the physical activity levels of PHCWs, with low engagement in moderate activity during work and limited active commuting. This study underscores the need for workplace wellness interventions, such as walking meetings and fitness facilities, to boost physical activity among healthcare workers, enhancing their health, resilience, and the quality of care they provide.

## 1. Introduction

Physical activity (PA) spans a spectrum of bodily movements, from the rhythmic strokes of swimming to the energetic stride of jogging, encompassing actions that expend energy and enhance health by improving cardiovascular fitness and mental health [1,2]. Its intensity, categorised as mild, moderate, or vigorous, is determined by the physiological effort it demands, such as increases in heart rate and respiration [3]. For example, brisk walking exemplifies moderate-intensity activity, whereas competitive sports or running signify vigorous activity due to their substantial energy requirements. According to the World Health Organization (WHO) [4], adults should engage in at least 150 min of moderate or 75 min of vigorous activity per week, emphasising the importance of tailoring physical activity to individual preferences and lifestyles for sustainability.

Regular physical activity remains fundamental to health, acting as a preventive measure against chronic conditions including cardiovascular disease, diabetes, and hypertension while alleviating stress, anxiety, and depression through the release of endorphins and the reduction in cortisol levels [5]. Despite these well-documented benefits, physical inactivity remains a pressing global health issue [6], with approximately 31% or 1.8 billion adults failing to meet the recommended levels of PA necessary to maintain optimal health [7]. The highest prevalence of physical inactivity is observed in the high-income Asia–Pacific region, followed closely by South Asia, while the lowest rates are found in Oceania and sub-Saharan Africa [8]. In South Africa, a study involving 26,339 participants revealed that 57.4% were physically inactive, 14.8% engaged in moderate physical activity, and only 27.8% reported vigorous activity levels [9].

Primary healthcare workers (PHCWs) play a pivotal role in addressing these challenges as both caregivers and health advocates. Their physical activity patterns and lifestyle habits are crucial not only for their own well-being but also for their effectiveness as role models who influence public health behaviours [10]. Occupational physical activity, which encompasses job-related movements, varies significantly across professions depending on the nature and demands of the work [11]. In healthcare, nurses often demonstrate high levels of occupational physical activity, constantly moving to attend to patients and perform their duties [12]. Healthcare workers, despite their high energy expenditure and dynamic work environments, often face significant challenges such as musculoskeletal pain, fatigue, and stress [13]. These issues can negatively impact their work efficiency and overall health. The physical demands of their roles, including repetitive movements, long shifts, and strenuous tasks, further contribute to these risks [14]. While HCWs are tasked with promoting healthy behaviours among patients, their ability to serve as credible role models is strongly tied to their own adherence to active and healthy lifestyles. This dual role highlights the importance of understanding the PA patterns, lifestyle habits, and demographic characteristics of HCWs, as these factors can influence their capacity to deliver high-quality care and advocate for public health. Engaging in regular physical activity can help mitigate these risks by improving muscle strength, reducing pain, and enhancing job performance [15]. Adopting proper body mechanics, such as lifting with bent knees and a straight back and sitting with their feet flat on the floor and their shoulders aligned, taking regular breaks, and adhering to ergonomic principles are essential to protecting their musculoskeletal health [16].

Incorporating physical activity into healthcare workers’ routines also enhances mental well-being by reducing stress and improving mood. Exercise helps alleviate symptoms of anxiety, depression, and burnout by releasing endorphins and lowering cortisol levels [17]. Studies show that healthcare workers who engage in physical activity experience less stress, greater job satisfaction, and better emotional stability, which leads to a more effective and resilient workforce [18,19]. Moreover, workplace wellness initiatives, such as on-site fitness programmes, stress management workshops, and health education, can enhance healthcare workers’ physical and mental well-being while fostering a culture of health within healthcare organisations [20,21].

The WHO aims to achieve a 15% global reduction in physical inactivity by 2030, highlighting the urgency of addressing sedentary lifestyles to improve public health outcomes [8]. Assessing physical activity levels among healthcare workers (HCWs) is vital in achieving this target, as HCWs serve as key influencers of health behaviour within communities. HCWs’ engagement in physical activity can significantly impact the broader population’s activity levels. Furthermore, physical inactivity poses a substantial economic burden on public health systems, with costs estimated to reach USD 300 billion annually [22]. For example, treatment expenses for conditions such as coronary artery disease and type 2 diabetes include hospitalisation, outpatient services, and medications. Investing in interventions to promote physical activity, such as exercise programmes, can reduce these costs and offer long-term savings. Prioritising active lifestyles is essential to mitigate the economic burden on public health systems.

Healthcare professionals play a pivotal role in promoting regular physical activity by prescribing and encouraging it among patients to enhance health outcomes and aid in the prevention and management of chronic diseases such as diabetes, cardiovascular disease, and hypertension [23]. However, the effectiveness of these recommendations is closely linked to healthcare workers’ own adherence to an active lifestyle. Understanding the physical activity patterns of all HCWs, regardless of age, gender, or profession, is essential, not only to assess their contribution to global health targets but also to reinforce their credibility as advocates for healthier, more active communities. When all professionals within healthcare facilities prioritise physical activity, it not only enhances their own well-being and job performance but also strengthens their role in influencing positive health behaviours among the populations they serve [24]. This collective commitment to an active lifestyle is crucial for fostering a healthier workforce and improving overall public health outcomes.

The well-being of healthcare professionals is intricately linked to their ability to deliver high-quality patient care [24]. Understanding the relationship between PA, lifestyle habits, and demographic characteristics among PHCWs is therefore essential for identifying gaps and designing interventions tailored to their unique needs. Despite HCWs’ critical roles, there is limited literature examining the specific PA patterns of PHCWs, particularly in low- and middle-income countries like South Africa. Exploring these patterns can provide valuable insights into how occupational demands, cultural factors, and organisational policies influence their physical activity levels. For instance, cultural norms, particularly regarding gender roles, may shape participation in physical activity, while the absence of workplace wellness programmes or incentives for active commuting may further exacerbate sedentary behaviours [25]. Addressing these factors is key to promoting a healthier, more resilient healthcare workforce.

Evaluating physical activity among HCWs offers critical insights into how their behaviours align with global health objectives of reducing physical inactivity. Interventions targeting HCWs’ physical activity can serve dual purposes: improving their personal well-being and enhancing their influence as health advocates, thereby creating a ripple effect that supports the WHO’s global agenda for reducing physical inactivity and mitigating the financial strain on healthcare systems. The present study aims to assess the physical activity levels and lifestyle habits among primary healthcare providers in Lepelle-Nkumpi Municipality, located in South Africa’s Limpopo Province. The hypothesis for this study was that the PHCWs in Lepelle-Nkumpi Municipality have low levels of physical activity, influenced by demographic factors (such as gender, age, and occupational roles) and workplace-related conditions (such as sedentary job roles and lack of wellness programmes). By providing a detailed analysis of PA patterns and their implications, this study contributes to the evidence base necessary for designing targeted interventions that promote active lifestyles among healthcare workers. Such efforts can improve not only their personal health outcomes but also their capacity to deliver high-quality care and serve as credible advocates for public health.

## 2. Methods

### 2.1. Research Method and Design

This study employed a quantitative approach and a cross-sectional descriptive design, enabling a systematic assessment of occupational and leisure-time physical activity patterns among healthcare workers. The quantitative method allowed for the collection and analysis of numerical data, facilitating objective measurements of physical activity levels and sociodemographic characteristics. The cross-sectional design provided a snapshot of physical activity behaviours at a single point in time, capturing simultaneous measurements of sociodemographic factors and physical activity scores. This design was particularly effective in identifying patterns, associations, and potential disparities within the study population. Furthermore, it enabled a robust exploration of the relationships between key variables, offering valuable insights into factors influencing physical activity engagement among healthcare workers. By highlighting prevalent trends and correlations, this approach establishes a foundation for developing targeted interventions to promote physical activity within healthcare settings. The findings can inform the design of workplace wellness programmes, policy recommendations, and behaviour change strategies to enhance physical activity levels and overall well-being among healthcare professionals. The cross-sectional design identifies associations but cannot establish causality. It also fails to capture variations over time due to seasonal changes or workload fluctuations. Future longitudinal or experimental studies are needed for stronger causal inferences.

### 2.2. Study Setting and Participants

The research was conducted in the Lepelle-Nkumpi sub-district of the Capricorn district in Limpopo Province, South Africa. This sub-district comprises 23 fixed clinics, 7 mobile clinics, and 3 hospitals (Lebowakgomo, Magatle, and Thaba-Moopo Psychiatric Hospital), serving diverse regions including Zebediela, Mphahlele, Mathabatha, and Mafefe. The healthcare workforce in this area consists of professionals from various disciplines, with nurses comprising the majority. However, it also includes allied health professionals, support staff, and administrative personnel, representing a diverse range of ages and genders. This diversity ensures that the study provides a comprehensive assessment of physical activity levels and lifestyle habits across different professional roles and demographic backgrounds. Healthcare workers influence public health through their lifestyle choices. Understanding their physical activity patterns supports workplace wellness programmes and promotes healthier behaviours in the communities they serve. This study contributes to preventative healthcare and community health promotion efforts.

The study enrolled 174 participants, comprising healthcare workers employed at primary healthcare facilities within the sub-district. The inclusion criteria encompassed all healthcare workers aged 18 years or older, regardless of gender, who were permanently employed at these facilities and had provided informed consent to participate. The exclusion criteria applied to healthcare workers who were on prolonged leave or unable to participate due to medical conditions, ensuring that all participants could actively engage in the study assessments without limitations. By focusing on the collective well-being of healthcare providers, the study underscores the broader impact on the public they serve, highlighting the interconnectedness of workforce health and community health outcomes.

To enhance representativeness, stratified random sampling was employed, ensuring proportional representation across different professional categories, including nurses, allied health professionals, and support staff. This approach minimised sampling bias and allowed for a more comprehensive assessment of physical activity levels within the PHC workforce. Although the study sample was initially set at 174 participants, an additional 10% of the targeted sample was invited, resulting in a total of 191 eligible healthcare workers. This led to a response rate of 98%. The sample size for this study was determined using the Taro Yamane formula [26], $n=\frac{N}{1+N{\left(0.05\right)}^{2}}\;,\;$ where *n* = sample size, N = population size (N=), e = error margin (5%), sample size = 331/1 + 331 (0.05)^2^, *n* = 174.

### 2.3. Instrument

A structured, closed-ended questionnaire was employed, comprising three sections: demographic information, occupational physical activity, and leisure-time physical activity. The demographic section collected details on participants’ age, gender, marital status, professional category, and job title. The physical activity sections assessed the type, intensity, and duration of activities performed during work and leisure. The moderate and vigorous intensity levels were determined in alignment with WHO [5] guidelines.

The detection method used in this study was validated through a multi-step process to ensure accuracy, reliability, and applicability in assessing physical activity levels and lifestyle habits among healthcare workers. It was subject to expert validation by physiotherapists and research supervisors, focusing on content accuracy, relevance, and construct validity. Expert validation is a widely recognised method in questionnaire development and has been demonstrated to improve instruments’ relevance and effectiveness in similar populations [27,28,29]. In addition to expert validation, a pilot study was conducted to assess the tool’s reliability in a real-world setting. The pilot study involved 12 participants from a comparable primary healthcare clinic, where the questionnaire was tested for clarity, consistency, and applicability to the target population. The results confirmed the questionnaire’s reliability, with no issues in interpretation or response. No modifications were needed after the pilot.

To further confirm the instrument’s internal consistency, Cronbach’s alpha was calculated, yielding a value of 0.70, which is considered acceptable for reliability in social and health sciences research. This statistical verification ensures that the questionnaire consistently measures the intended constructs across various respondents, enhancing its reliability for assessing physical activity and lifestyle habits in healthcare workers.

#### Data Analysis

Data were systematically coded and analysed using the Statistical Package for Social Sciences (SPSS) version 28. Descriptive statistics were employed to summarise demographic variables and physical activity patterns, providing a structured overview of participant characteristics. Occupational physical activity was assessed in terms of engagement duration and adherence to recommended activity levels (30 min or more per workday). Leisure-time physical activity was analysed to determine the proportion of participants engaging in moderate- or vigorous-intensity activities, as well as their frequency of regular exercise.

Participant responses were converted into habits and lifestyle scores on a scale of 0–100%, categorised as poor (0–50%), good (51–80%), and excellent (81–100%). Associations between lifestyle scores and demographic variables such as gender and job title were examined using chi-square tests, with statistical significance set at *p* < 0.05. Chi-square tests were selected for analysing categorical data and assessing associations between demographic variables and physical activity outcomes. Unlike logistic regression, which predicts binary outcomes, chi-square tests are ideal for examining relationships between categorical variables (e.g., gender, job title) and activity levels (e.g., poor, good, excellent), without requiring assumptions about the data distribution. Logistic regression would be more appropriate for predicting specific outcomes, but the chi-square test was better suited to this study’s objectives of evaluating associations. To enhance clarity, occupational physical activity classifications were defined as follows: Walking for a very short time refers to walking for less than 10 min per session as part of routine occupational tasks. Mostly sitting indicates spending 75% or more of the workday seated, with little to no physical movement. Mostly standing describes spending a significant portion of the workday (50–75%) on one’s feet but without performing any notable physical activity. Mostly doing moderate activity involves performing tasks that require moderate physical effort, such as lifting, moving items, or brisk walking, for a continuous duration of 10 to 30 min per session.

### 2.4. Ethical Issues

Ethical approval for the study was granted by the Turfloop Research Ethical Committee (TREC) at the University of Limpopo, under clearance certificate number TREC/586/2022:PG. Permission to conduct research within healthcare facilities was obtained from the Limpopo Department of Health (reference: LP 202301-017).

Prior to participation, informed consent was obtained from all participants, ensuring they fully understood the study’s objectives, procedures, and their voluntary participation rights. Participants were explicitly informed of their right to withdraw from the study at any stage without any consequences. To uphold ethical integrity, strict measures were implemented to safeguard privacy and maintain confidentiality. All participant data were anonymised and securely stored, ensuring compliance with ethical research standards and data protection protocols throughout the study.

## 3. Results

In this study, 174 individuals participated, as indicated in Table 1, which details the sociodemographic profile of the participants. This study consisted of a majority of females (89%) and a minority of males (11%), with a notable portion (66%) of participants aged 40 years or older. Moreover, only 48% of the participants reported being married. In terms of professional roles, the majority were nurses (64%), followed by support staff (31%) and allied healthcare professionals (5%).

**Figure 1:**
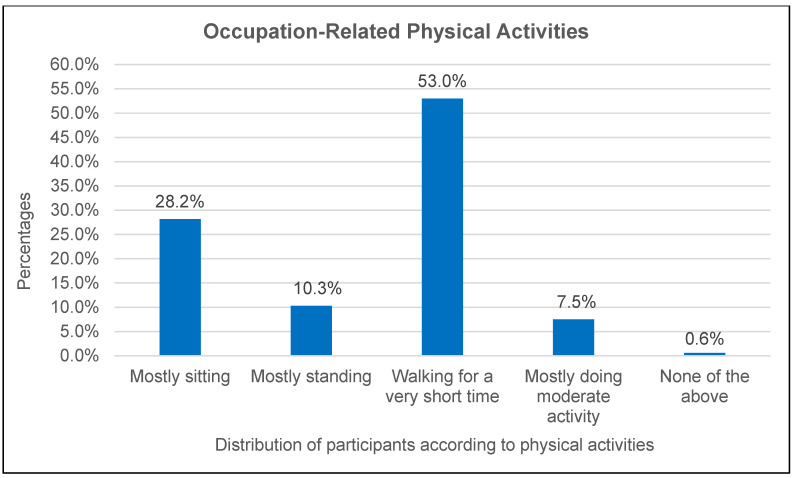
Occupation-related physical activities.

Figure 1 depicts the distribution of participants’ occupation-related physical activities. It shows the percentage of participants engaging in different physical activities related to their occupation. The majority (53.0%) reported “walking for a very short time”, followed by 28.2% who spend their time “mostly sitting” and 10.3% who are “mostly standing”. A smaller proportion (7.5%) reported “mostly doing moderate activity”, while a very small percentage (0.6%) reported doing “none of the above”. These findings indicate limited engagement in activities that meet recommended intensity levels for health benefits, such as moderate-to-vigorous physical activity.

Table 2 presents the distribution of participants’ engagement in physical activities. Among the participants, 47.1% indicated no involvement in vigorous activities, while 39.7% allocated 60 min or less and 13.2% allocated 30 min or less to such activities. In terms of moderate activity, 56.3% refrained entirely, while 25.3% dedicated less than 60 min, and 18.4% dedicated 30 min or less. When considering the frequency of vigorous activities per week, 82% reported 1–3 days, with 17.8% reporting 4–7 days. Similarly, for moderate-intensity activities, 93% reported 1–3 days, while 6.9% reported 4–7 days. These data suggest sporadic physical activity patterns that fall short of WHO guidelines.

Table 3 presents the distribution of participants’ physical activity related to travel. It reveals that 59.8% of the participants do not participate in walking or cycling for transportation, while 45.4% reported spending 30 min on traveling on an average day.

Table 4 illustrates the breakdown of participants’ engagement frequencies in non-work-related and leisure-time physical activities. It shows that 64.4% of the participants are involved in vigorous- or moderate-intensity physical activity during their leisure time, whereas only 33.9% engage in such activities during their free time. Additionally, only 37.9% of the participants reported spending 30 min or more on these activities on a typical day. Regarding the frequency of engagement in vigorous activities during leisure time, 85% of the participants indicated 1–3 days per week, while 15% exercised 4–7 days per week. This result highlights a missed opportunity to incorporate active commuting into daily routines.

**Figure 2:**
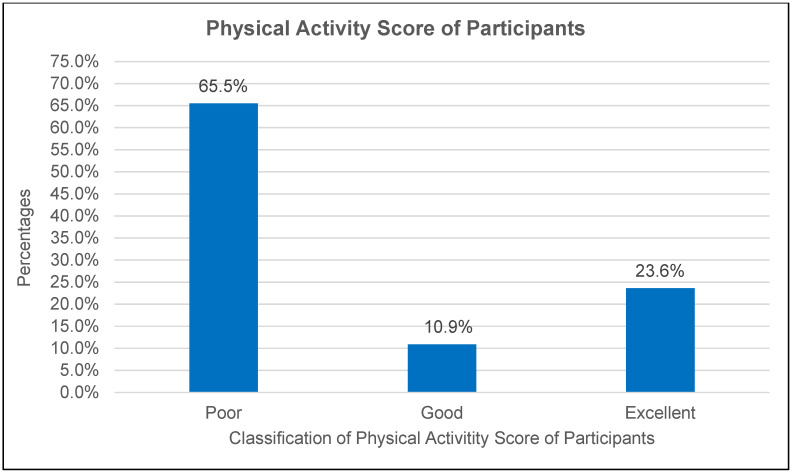
Physical activity scores of participants.

Figure 2 depicts the distribution of habit and lifestyle scores among the participants, showing that 65.5% had poor scores (1–50%), 10.9% had good scores (51–80%), and 23.6% had excellent scores (81–100%). These findings underscore a lack of consistent leisure-time physical activity, which could serve as a compensatory mechanism for limited occupational activity.

Table 5 shows that gender was the sociodemographic variable significantly associated with lifestyle habits (*p* = 0.022).

## 4. Discussion

The primary objective of this study was to assess physical activity levels and lifestyle habits among primary healthcare workers in Lepelle-Nkumpi Municipality, Limpopo Province, South Africa. This study confirms the hypothesis that PHCWs in Lepelle-Nkumpi Municipality have low physical activity levels, influenced by workplace conditions and demographics. These findings were expected due to demanding schedules, high workloads, and limited wellness programmes [11,14]. This study underscores the need for workplace health initiatives, such as integrating physical activity into routines and promoting active commuting. The results contribute to the occupational health literature and are valuable for healthcare administrators and policymakers in designing strategies to improve PHCWs’ well-being, ultimately supporting public health goals. This study’s demographic profile reveals that out of the 174 participants, only 20 were men (11%), reflecting the broader gender distribution within healthcare settings, particularly in primary healthcare. This gender disparity is not unique to this study but aligns with national workforce trends. According to South African Nursing Council statistics, only 10.4% of practising nurses in South Africa are male, with male nursing students comprising just 8% of enrolments [30]. This highlights a systemic underrepresentation of men in primary healthcare professions. Despite the lower male participation, this study was intentionally designed to assess physical activity patterns and lifestyle habits among PHCWs as a collective workforce, rather than focusing on gender-specific trends. However, the inclusion of men remains critical, as their health behaviours, occupational demands, and participation in wellness initiatives contribute to a comprehensive understanding of workplace well-being. Moreover, healthcare workers serve as role models for the public, influencing community perceptions, health-seeking behaviours, and attitudes toward wellness. A well-supported and health-conscious workforce can drive positive public health outcomes, reinforcing the importance of inclusive health promotion strategies [31]. The data reveal that walking constitutes the most common form of occupational physical activity, with 53% of participants engaging in walking for shorter durations during work hours. This finding reflects the nature of healthcare work, where tasks such as attending to patients and moving between departments inherently involve walking [32]. However, only 7.5% of the participants reported engaging in moderate-intensity activities during work hours, a level of activity essential for cardiovascular health and chronic disease prevention [33]. This underrepresentation of moderate and vigorous physical activity points to a critical gap in integrating meaningful exercise into occupational routines.

Nearly half (47.1%) of the participants reported no engagement in vigorous physical activity at work, highlighting the sedentary aspects of healthcare professions. Contributing factors may include long working hours, high patient loads, and limited opportunities for breaks, which discourage engagement in physical activity during work [34,35]. Furthermore, cultural norms and attitudes toward physical activity, especially in the context of gender roles, may also influence participation rates, as highlighted by the significant gender disparities in lifestyle scores (*p* = 0.022). The lack of organisational policies promoting active work environments, such as incentives for active commuting or on-site fitness programmes, likely exacerbates these sedentary behaviours [36]. Addressing these barriers through workplace and policy interventions could significantly enhance physical activity levels. Interventions such as walking meetings, active breaks, and ergonomic modifications to promote movement could counteract this trend [37,38].

Encouragingly, 64.4% of the participants reported engaging in moderate-to-vigorous physical activity during their leisure time. However, the consistency of participation remains a concern, with only 33.9% engaging in these activities regularly. Regular physical activity is essential for sustained health benefits, including improved cardiovascular function, enhanced mental health, and reduced stress [39,40]. These findings align with previous studies highlighting the challenges of maintaining consistent physical activity levels among healthcare workers. For example, a study found that while many healthcare workers engage in physical activity, the frequency and duration often fall short of recommended levels, limiting the long-term health benefits [41]. Additionally, only 37.9% of the participants spent 30 min or more on leisure-time physical activities daily, which is below the WHO’s recommended 150 min per week [4]. This finding is consistent with research which noted that a significant portion of healthcare workers fail to meet the recommended activity levels, despite recognising the importance of physical activity for overall well-being [42].

These findings underscore the need for targeted interventions to promote both consistency and longer-duration physical activity during leisure time. Interestingly, while 85% of the participants engage in vigorous activities 1–3 times per week during their leisure time, increasing this frequency could provide additional health benefits. This observation is supported by a study which found that a higher frequency of vigorous physical activity was associated with improved health outcomes, including a reduced risk of chronic diseases [43]. Structured wellness programmes that emphasise the importance of regularity and progression in physical activity levels could be highly impactful, potentially helping healthcare workers meet the recommended activity levels and achieve sustained health improvements.

Regular physical activity has been extensively documented as a key factor in stress reduction, which is particularly crucial for healthcare professionals who often experience high levels of occupational stress due to demanding work environments. Physical activity triggers the release of endorphins and reduces cortisol levels, improving mood and fostering mental resilience [44]. This enhanced mental well-being positively impacts work efficiency by enabling healthcare workers to maintain focus, manage fatigue, and handle patient care more effectively. Furthermore, physical activity fosters social interactions, teamwork, and a sense of community among colleagues, improving workplace dynamics and overall well-being [43]. These benefits underline the importance of workplace wellness initiatives that encourage physical activity to mitigate burnout, reduce turnover, and promote a healthier workforce.

The findings from Figure 2 underscore a concerning prevalence of poor habits and lifestyle choices among PHCWs, with 65.5% of the participants exhibiting poor scores. This aligns with the existing literature emphasising the challenges healthcare workers face in maintaining healthy lifestyles due to long hours, high job demands, and limited access to healthy options [45,46,47]. Poor lifestyle habits among PHCWs are particularly problematic, as they can lead to chronic diseases, burnout, and compromised patient care quality.

Conversely, the observation that 23.6% of the participants demonstrated good or excellent lifestyle scores is encouraging. This suggests that despite the inherent challenges of healthcare work, many PHCWs adopt strategies to maintain healthier habits. These findings underscore the need to identify and amplify successful strategies among this group. For instance, workplace wellness initiatives, such as healthy food programmes, accessible fitness facilities, and stress reduction workshops, have shown promise in improving lifestyle behaviours among healthcare workers [48,49,50].

The findings from Table 5 reveal that while age and marital status did not significantly influence habits and lifestyle choices, gender was an important determinant (*p* = 0.022). These results are consistent with previous research, which highlights disparities in physical activity levels and dietary behaviours across genders and professional roles within healthcare settings [51,52,53]. Female healthcare workers often face challenges in engaging in regular physical activity due to balancing professional responsibilities with caregiving roles at home, leading to time constraints, stress, and lower prioritisation of well-being. In contrast, male healthcare workers may face fewer household-related barriers [54]. Additionally, physically demanding or irregular job schedules, particularly in nursing, can further hinder physical activity for both genders. Interventions addressing gender-specific barriers and the challenges of demanding job roles are essential for promoting healthy behaviours and improving health outcomes among healthcare workers. The significant association between gender and lifestyle activity scores highlights a need for gender-sensitive interventions. Programmes should target men to address barriers limiting their engagement in healthy habits. Age and marital status do not significantly influence activity scores, suggesting that workplace-specific and gender-related factors may be more critical in determining habits and lifestyles. Female PHCWs, often balancing professional and personal responsibilities, may face unique challenges in incorporating physical activity into their routines. Similarly, job roles with higher physical demands or irregular schedules, such as nursing, may negatively impact lifestyle choices.

The findings highlight the need for targeted interventions to address the low levels of moderate and vigorous physical activity and poor lifestyle scores among PHCWs. Strategies should include the following:

- Workplace policies: Establish policies that promote active commuting, walking meetings, and on-site fitness facilities, with incentives to encourage physical activity during work hours. Ergonomic improvements could help mitigate sedentary behaviour.
- Gender-sensitive interventions: Tailor programmes to address the unique challenges faced by female PHCWs, such as balancing family and work responsibilities, while also encouraging male PHCWs to engage in wellness initiatives.
- Wellness programmes: Implement comprehensive wellness initiatives, offering support for physical activity, nutrition, and mental health. These programmes should include subsidised healthy meals and accessible fitness resources, ensuring inclusivity for both genders and all job roles.
- Personalised interventions: Develop tailored programmes accounting for gender-specific and job-related challenges to ensure inclusivity and effectiveness.

In resource-constrained settings like South Africa, the implementation of wellness programmes may encounter difficulties due to financial and infrastructural limitations. However, cost-effective and practical solutions, such as mobile fitness programmes or partnerships with local fitness centres, could serve as viable alternatives to enhance the impact of wellness interventions. This could involve the South African government promoting or introducing policies that encourage active commuting or providing infrastructure to facilitate physical activity. Furthermore, future research should investigate the role of organisational culture in influencing physical activity behaviours and examine how socio-economic and cultural factors affect participation in wellness initiatives. Longitudinal studies and the use of objective tools, such as wearable activity trackers, would offer more robust and reliable data for developing evidence-based interventions.

This study identifies critical gaps in understanding the interplay between occupational and leisure-time physical activity and lifestyle behaviours. Future research should explore longitudinal data to establish causal relationships between workplace dynamics, physical activity levels, and health outcomes. Investigating the influence of socio-economic and cultural factors on physical activity patterns among PHCWs could also provide valuable insights for designing context-specific interventions.

While this study presents insightful data, several limitations must be considered. Notably, the influence of social, cultural, and contextual factors on the physical activity and lifestyle behaviours of PHCWs was not explored in depth. These factors may offer additional explanatory power regarding why certain behaviours are more prevalent in specific groups. Furthermore, this study did not use standardised instruments, such as the International Physical Activity Questionnaire (IPAQ) or the Global Physical Activity Questionnaire (GPAQ), which could have provided more consistent and comparable data on physical activity levels. However, the study’s data collection instrument was validated, and its reliability was confirmed through pilot testing. Future studies should integrate these standardised tools and consider a broader range of sociocultural factors. This study captured data at a single point in time, limiting its ability to establish causal relationships between physical activity patterns and health outcomes. Future research employing longitudinal designs could provide more robust insights into changes over time and the impact of interventions than a cross-sectional study. Data were collected through self-reported questionnaires, which are subject to recall bias, social desirability bias, and inaccuracies in reporting physical activity levels. Incorporating objective measures, such as wearable activity trackers, in future studies could enhance accuracy and reliability.

## 5. Conclusions

This study highlights significant gaps in the physical activity levels and lifestyle habits of primary healthcare workers (PHCWs) in Lepelle-Nkumpi Municipality, with a notably low prevalence of moderate physical activity during work hours. The findings indicate a high proportion of sedentary behaviour, with limited engagement in structured physical activity, particularly during working hours. Additionally, the low rates of active commuting, such as walking or cycling to work, further underscore the need for interventions to enhance daily physical activity levels.

To address these disparities, the implementation of targeted workplace wellness programmes is essential. These should include structured physical activity initiatives, such as walking meetings, active breaks, and on-site fitness facilities, to encourage movement throughout the workday. Additionally, promoting active commuting through infrastructural support and incentives can help integrate physical activity into daily routines. Culturally tailored health campaigns should also be developed to address barriers to physical activity specific to healthcare settings and local communities.

Furthermore, this study provides valuable insights into the link between workplace environments, physical activity, and overall healthcare service delivery. Given the physically and mentally demanding nature of healthcare professions, integrating wellness-focused interventions within healthcare settings is critical to fostering a resilient and capable workforce. These findings serve as a foundation for developing evidence-based policies and workplace programmes that prioritise health and well-being among PHCWs. Ensuring a healthy and active workforce is not only vital for their own well-being but also enhances their capacity to deliver high-quality patient care and serve as credible advocates for public health.

## Figures and Tables

**Table 1:** Demographic profile of participants, *n* = 174.

Demographic Profile		Number of Participants	Percentages of Participants
Gender	Female	154	89%
	Male	20	11%
Age	≤40 years	60	34%
	>40 years	114	66%
Marital status	Unmarried	91	52%
	Married	83	48%
Race	African	174	100%
Profession	Nursing	112	64%
	Allied	8	5%
	Support staff	57	31%

**Table 2:** Frequency distribution of participants according to level of vigorous and moderate activities, *n* = 174; % in rows.

Statements	Minutes		0 min
	30 min or Less	More than 30 min	
Daily time spent on vigorous activities	23 (13.2%)	69 (39.7%)	82 (47.1%)
Daily time spent on moderate activities	32 (18.4%)	44 (25.3%)	98 (56.3%)
Daily time spent on exercise activities	3 (1.7%)	171 (98.3%)	0 (0%)
**Statements**		**Days**	
		**1–3 Days**	**4–7 Days**
Frequency of engaging in vigorous activities, weekly		143 (82.2%)	31 (17.8%)
Frequency of engaging in moderate-intensity activities, weekly		162 (93%)	12 (6.9%)

**Table 3:** Frequency distribution based on participants’ travel-related physical activity; total participants *n* = 174, with percentages represented in rows.

Statements		Yes	No
Do you engage in walking or bicycling to travel to and from different locations?		70 (40.2%)	104 (59.8%)
	More than 30 min	(30 min or less)	None
How much time do you typically dedicate to walking or cycling for transportation on a regular day?	47 (27%)	79 (45.4%)	48 (27.6%)
**Statement**		**Days**	
		**1–3 days**	**4–7 days**
During a typical week, how frequently do you engage in walking or cycling to travel to and from different places?		148 (85%)	26 (15%)

**Table 4:** Distribution of participants’ frequencies of non-work-related and leisure-time physical activity, *n* = 174; % in rows.

Statements		Yes	No
Do you engage in any moderate-intensity or vigorous physical activity during your leisure or free time?		59 (33.9%)	112 (64.4%)
**Statements**	**Minutes**		
	**30 min or less**	**More than 30 min**	**None**
On a typical day, how much time do you allocate to this activity?	66 (37.9%)	43 (24.7%)	65 (37.4%)
**Statement**		**Days**	
		**1–3 days**	**4–7 days**
During a typical week, how often do you engage in vigorous activities during your leisure or spare time?		148 (85%)	26 (15%)
		**Minutes**	
		**More than 30 min**	**30 min or less**
In the past week, how many hours did you spend sitting or reclining (excluding sleeping) on a typical day?		135 (77.6%)	39 (22.4%)

**Table 5:** Association between sociodemographic profile and habits and lifestyle activities.

Sociodemographic Profile	Chi-Square (X^2^) Value	*p*-Value	Interpretation
Gender	19.336	0.022	Statistically significant association between gender and lifestyle habits.
Age	17.833	0.467	No significant association between age and lifestyle habits.
Marital status	7.172	0.619	No significant association between marital status and lifestyle habits.
Professional category	22.001	0.232	No significant association between professional category and lifestyle habits.

## Data Availability

This article is based on the data collected from primary healthcare workers in Lepelle Nkumpi Municipality in Limpopo Province, South Africa. Therefore, the data generated or analysed during the current study are not publicly available but can be made available through request, which can be submitted to the corresponding author.
